# 

**Published:** 1876-11

**Authors:** 


VOL. 23. NOVEMBER, 1876. No. 11.

TWENTY-THIRD YEAR.

### HALL'S
### Journal of Health.

PUBLISHED AT 137 EIGHTH STREET, NEW YORK,

**Four Doors East of 756 Broadway.**

COPYRIGHT 1876, BY E. H. GIBBS.

AGENTS FOR THE TRADE:

AMERICAN NEWS COMPANY, 121 NASSAU STREET.

NEW YORK NEWS COMPANY, 18 BEEKMAN STREET.

**Terms, $2 a Year.**

**SINGLE NUMBER, 20 CENTS.**

JOHN KENT, Printer, 11 Frankfort St., New York.

